# Intraperitoneal administration of follistatin promotes adipocyte browning in high-fat diet-induced obese mice

**DOI:** 10.1371/journal.pone.0220310

**Published:** 2019-07-31

**Authors:** Haoyu Li, Chuanhai Zhang, Junyu Liu, Wenya Xie, Wentao Xu, Fei Liang, Kunlun Huang, Xiaoyun He

**Affiliations:** 1 Beijing Advanced Innovation Center for Food Nutrition and Human Health, College of Food Science and Nutritional Engineering, China Agricultural University, Beijing, China; 2 Key Laboratory of Safety Assessment of Genetically Modified Organism (Food Safety), Ministry of Agriculture, Beijing, China; 3 Department of Reproductive Physiology, Zhejiang Academy of Medical Sciences, Hangzhou, China; University of Texas Health Science Center at Houston, UNITED STATES

## Abstract

With rapid economic development, the prevalence of obesity has increased remarkably worldwide. Obesity can induce a variety of metabolic diseases, such as atherosclerosis, diabetes, hypertension and coronary heart disease, which significantly endanger the health and welfare of individuals. Brown and beige fat tissues play an important role in thermogenesis in mammals. Recent studies have shown that follistatin (FST) can potentially induce the browning of white adipose tissue (WAT). In this study, high-fat diet-induced obese mice were injected with follistatin for one week to explore the effects of follistatin on browning and metabolism and to determine the mechanism. The results showed that follistatin suppressed obesity caused by a high-fat diet and increased insulin sensitivity, energy expenditure, and subcutaneous fat browning. The beneficial effects remained even after a period of withdrawal. Follistatin promoted secretion of irisin from subcutaneous fat via the AMPK-PGC1α-irisin signal pathway, which induces browning of WAT, and activated the insulin pathway in beige fat thereby promoting metabolism.

## Introduction

Brown adipose tissue (BAT) plays an important role in non-shivering thermogenesis, especially in the newborns [1]. BAT is different from WAT with respect to energy metabolism. WAT stores energy mainly in the form of triglycerides during periods of excess energy intake, where BAT releases heat by promoting the consumption of triglycerides and glucose [2]. BAT is characterized by fat accumulation in the form of multilocular lipid droplets, and the presence of numerous mitochondria containing a unique thermogenic protein named uncoupling protein 1 (UCP1). UCP1 is abundantly expressed in the mitochondria of mammalian BAT, and can burn chemical fuels by uncoupling cellular respiration to defend against obesity [3].

When white adipocytes are stimulated by specific factors, such as cold or β3-adrenoceptor, the size of white adipocytes shrinks, the heat production increases, and the adipocytes begin browning into beige cells [4]. Beige adipose tissue is similar to BAT in morphology and function, as it consists of multilocular lipid droplets and contains a large number of mitochondria. In addition to UCP1, other key BAT-selective genes are highly expressed, such as *Prdm16*, *Pgc1α*, and *Pparγ*. These BAT-selective proteins can promote lipid metabolism and increase the energy expenditure [5, 6]. It is well established that BAT and beige fat can improve insulin sensitivity and decrease body weight in humans and experimental animals. In addition, high levels of the CD137 and TMEM26 proteins are expressed in beige fat, which are key selective markers in beige adipose tissue.

FST is a secreted glycoprotein, which has a high cysteine content. FST has a strong affinity for follicle-stimulating hormone (FSH), activin, and TGF-β superfamily members. FST binds to these proteins in an nearly irreversible manner, making them unable to activate their own receptors [7, 8]. FST is produced in a variety of tissues and organs in adult mammals through autocrine and paracrine mechanisms. Recently, a number of studies have reported that FST can activate BAT and promote the browning of WAT [9, 10]. Myostatin (MST) knockout mice, which were antagonized by FST as a member of TGF-β superfamily, expressed BAT-selective proteins in WAT and showed the appearance of browning [11]. Similarly, in MST knockout mice, the translational levels of the thermogenic genes *Ucp1* and *Pgc1α*, and the transcriptional levels of the beige adipose tissue marker genes *Tmem26* and *Cd137* were increased [12]. Recently, a number of studies have demonstrated that there were higher levels of fatty acid oxidation and energy metabolism in WAT of MST knockout mice compared to that in wild-type mice[13, 14]. As an inhibitor of MST, exogenous FST increased the expression of thermogenic genes 50%–80% in differentiated preadipocytes. In FST knockout mouse embryonic fibroblasts (MEFs), the expression of the UCP1 protein dropped by 50% compared with wild-type cells [14, 15].

In FST overexpressing mice, BAT mass increased, and the expression of BAT-selective and beige-selective proteins in WAT increased, indicating the formation of beige adipose tissue. At the same time, the expression levels of pp38MAPK/pERK1/2 phosphorylation increased [16]. Inhibition of the pp38MAPK/pERK1/2 pathway in 3T3-L1 cells impedes FST-induced UCP1 protein upregulation, suggesting that FST may induce WAT browning via the pp38MAPK/pERK1/2 pathway. The browning of WAT was observed in MST-knockout mice, and this was shown to be via activation of the AMPK-PGC1α-Fndc5 pathway in muscle [12]. The fibronectin type III domain containing 5 (Fndc5), which is the precursor substance of irisin, acts as a muscle factor to promote the expression of brown fat and beige fat-related proteins. As a newly defined myokine, irisin can promote the expression of BAT-selective and beige-selective proteins [17]. In fact, Fndc5 is not only secreted in muscle but also in WAT [18]. As an inhibitor of MST, FST may induce browning of WAT through the AMPK-PGC1α-Fndc5 pathway [19].

At present, there are limited studies of FST in animals, which have used lentiviral vectors to construct an FST-transgenic model [16]. Although this model can stably express high levels of FST in mice for an extended period of time, the method is not suitable for exploring the medicinal value of FST in clinical use. Because FST is an autocrine and paracrine protein which is secreted into the blood by various of tissues and organs in mammals [20], it is feasible to improve blood FST levels within a short time to promote metabolism and regulate blood glucose homeostasis in mice by external injection of FST. In the current study, we show that one week of FST injection can increase metabolic levels and induce subcutaneous WAT browning, possibly by activating AMPK-PGC1α-Fndc5 and pp38MAPK/pERK1/2 pathways, and this effect was observed after stopping the injections for one week. The current work is valuable for the clinical application of FST in activating browning of WAT for the treatment of metabolic syndrome.

## Experimental section

### Animals

Four- to six-week-old C57BL/6J male mice were obtained from Vital River Laboratories (Beijing). The obesity model was induced using a high- fat diet (HFD), which contained 60% of the energy provided by fat. The experimental design was approved by the Animal Ethics Committee of China Agricultural University, Beijing, and the approval ID of this study was KY20170027. The study was performed in a Specific Pathogen Free animal room of the Supervision, Inspection and Testing Center for Genetically Modified Organisms of the Ministry of Agriculture (Beijing, China; license number SYXK (Beijing) 2015–0045). The animal room was well controlled as the air was exchanged 15 times in an hour, a 12-hour light-dark schedule was maintained, the temperature was controlled at 22 ± 2°C, and humidity was controlled at 55% ± 10%. The chow fed to experimental animals was purchased from Hufukang biotechnology company (Beijing). The composition and content of the HFD are shown in Table 1. The weight ratio and energy ratio of the experimental diets are shown in Table 2.

**Table 1:** Composition and content of HFD.

	HFD
Casein	25.845
Cystine	0.388
Corn starch	0.000
Maltodextrin	16.153
Sucrose	8.891
Cellulose	6.461
Bean oil	3.231
Lard	31.660
M1002	1.292
DCP	1.680
Calcium carbonate	0.711
Potassium citrate monohydrate	2.132
V1001	1.291
Choline bitartrate	0.258
Edible dye	0.007
total:	100.000

**Table 2:** Weight ratio and energy ratio HFD.

	HFD	
	Weight ratio g%	Energy ratio kcal%
Protein	26	20
Carbohydrate	26	20
Fat	35	60
total:		100
kcal/g	5.24	

### FST injection experiment

Before the experiment, all animals were allowed one-week acclimation to adapt to the conditions of the animal room, and then fed with high-fat diet for the next 8 weeks to induce obesity. After high-fat induction, 12 mice with similar body weights were divided into two groups: One group was intraperitoneally injected with 8.5μg/kg bodyweight of Recombinant Mouse Follistatin 288 (R&D systems, 769-FS-025) once a day for one week, while the other group was marked as the control.

### FST withdrawal experiment

To explore the persistence of the physiological effect of FST, another 18 high-fat diet-induced obese mice were divided into 3 groups. One group of mice was marked as the follistatin withdrawal group (FSTW group), which was intraperitoneally injected with 8.5μg/kg bodyweight of FST once a day for one week but had no injection treatment for the next week. The second group was intraperitoneally injected with the same volume of phosphate buffered saline (PBS) once a day for one week, and had no injection treatment for the next week, this was marked as the PBS withdrawal group (PBSW group). The third group was injected with FST the same as the first group and when the first week passed, the mice in this group were sacrificed and their inguinal subcutaneous WAT sampled for Western blotting analysis.

In all experiments, 3 mice in the same group were housed in one cage with free access to food and water. All animals were fed a high-fat diet and body weight was recorded once a week. At the end of the experiment, the mice were sacrificed and blood was collected. Inguinal subcutaneous WAT was fixed using 4% paraformaldehyde and frozen in liquid nitrogen.

### Rectal temperature and infrared imaging

The mice were placed in a cold room (4°C) for 4 h and their rectal temperature was measured by temperature sensor AT210 (Zhongyi Dapeng, Shenzhen, China). Then, photos of the animals were taken using a handheld infrared camera FLIR T600 (FLIR, Oregon, US) with a white board as the background.

### Activity and energy expenditure

At the end of the FST withdrawal experiment, the mice were placed in a six-cage activity meter. After a 12 h acclimation period, activity was measured for 24 h. During the measurement of the activity, the mice had access to the HFD and water.

At the end of the FST withdrawal experiment, the mice were placed in a six-cage oxygen consumption meter. After a 12 h acclimation period, oxygen consumption (VO_2_) and carbon dioxide production (VCO_2_) were measured for the next 24 h. The data were recorded and the respiratory exchange ratio (RER) and energy expenditure (EE) were calculated using the formula (RER = VCO_2_/ VO_2_, EE = VO_2_ × [3.815 + (1.23 × RER)] × 40.1868); the unit used was kj/kg/h [21, 22].

### Intraperitoneal glucose tolerance test

At the end of the experiment (the FST injection experiment finished in one week, and the FST withdraw experiment finished in two weeks), an intraperitoneal glucose tolerance test (GTT) was carried out. All animals were fasted for 16 h and then given an intraperitoneal injection of glucose (1.5g/kg body weight). Blood glucose levels were detected at 0, 15, 30, 60, and 120 min after the glucose injection using blood glucose meters (ACCU-CHEK, Performa) and the area under the curve (AUC) between 0 and 120 min was calculated.

### Homeostasis model assessment-insulin resistance

The levels of serum insulin were determined with an insulin ELISA kit (Beyotime, PI602). Homeostasis model assessment-insulin resistance was calculated using the formula [23]: concentration of blood insulin(μg/L)×concentration of blood glucose (mg/dL)/22.5; the concentration of blood glucose was measured at 0 min in the GTT.

### Hematoxylin and eosin staining and immunohistochemistry

The subcutaneous adipose tissue fixed in 4% paraformaldehyde was cut to 1.0 cm×1.0 cm×0.3 cm cakes and embedded in paraffin. Each embedded tissue was then sliced to 5 μm thickness, placed on slides, and stained by hematoxylin and eosin (HE). The cell morphology of subcutaneous adipose was observed under the microscope. For immunohistochemistry (IHC) staining, tissue sections were incubated with blocking buffer for 1 h after antigen retrieval. Then, the tissue sections were incubated overnight with diluent containing Ucp1 primary antibodies. After washing with TBST, the samples were incubated for 60 min with diluent containing secondary antibodies. Then, the tissue sections were washed and reacted with ECL reagent.

### Real time-qPCR analysis

Total RNA was extracted using Trizol. Frozen subcutaneous adipose (0.1 g) was added into 1ml Trizol and a handheld homogenizer was used to break up the tissue. Samples were centrifuged for 10 min at 12,000 g, 0.2 ml chloroform was added to the supernatant, and samples were centrifuged for 15 min at 12,000 g. The supernatant was then mixed with 0.5 ml isopropanol and centrifuged 10 min at 12,000 g. The sedimentation after centrifugation contained total RNA, which was collected for further analysis.

The inverse transcription of RNA was performed using the One Step gDNA removal and cDNA Synthesis Supermix kit (Transgen Biotech, AT311). The real time-qPCR (RT-qPCR) was carried out using the Green qPCR Supermix kit (Transgen Biotech, AQ101). The cyclophilin gene was used as an internal control to quantify the expression of the BAT maker genes and beige maker genes and all primers were synthesized by the Beijing Ruibiotech company. The primers used are shown in Table 3 [24–26].

**Table 3:** Primer sequences.

Primer Names	Primer Sequences
Prdm16-F	GAAGTCACAGGAGGACACGG
Prdm16-R	CTCGCTCCTCAACACACCTC
CyclophiA-F	CAAATGCTGGACCAAACACA
CyclophiA-R	GCCATCCAGCCATTCAGTCT
UCP1-F	GGCAAAAACAGAAGGATTGC
UCP1-R	TAAGCCGGCTGAGATCTTGT
PGC-1a-F	ACAGCTTTCTGGGTGGATTG
PGC-1a-R	TGAGGACCGCTAGCAAGTTT
Tmem26-F	ACCCTGTCATCCCACAGAG
Tmem26-R	TGTTTGGTGGAGTCCTAAGGTC
CD137-F	CGTGCAGAACTCCTGTGATAAC
CD137-R	GTCCACCTATGCTGGAGAAGG
PPARγ-F	TCGCTGATGCACTGCCTATG
PPARγ-R	GAGAGGTCCACAGAGCTGATT
COX2-F	CCTGGTGAACTACGACTGCT
COX2-R	GACGGCTCATGAGTGGAGAA
LHX8-F	GAGCTGGTATGTGACGAGCA
LHX8-R	GTGAGCGTCTTAGGCTCCAG
Zic1-F	CTGGTCAACCACATCCGAGT
Zic1-R	TTGCAAAGGTAGGGCTTGTC

### Western blotting

Total protein from subcutaneous adipose was extracted using RIPA lysis buffer containing 1 mM PMSF. The homogeneous mixture was centrifuged at 10,000 g and the protein concentration of the supernatant was detected using a BCA kit (Beyotime Biotechnology, P0011). The protein solution was then mixed with loading buffer and heated for 5 min in boiling water.

An equal amount of each protein sample was loaded and separated by 12.5% SDS PAGE in an electrophoresis tank. Then, the protein of sample was transferred from the gel to a PVDF membrane. The PVDF membrane carrying the protein was blocked with 5% BSA. Western blotting was carried out using corresponding detection antibodies.

### Statistical analysis

Data was presented as mean ± SEM, and differences between groups were analyzed by Student’s t-tests using GraphPad Prism 5.0. If *p* value of T-test was less than 0.05, the difference was considered as significant.

## Results

### FST injection decreased body weight in obese mice

**Fig 1:**
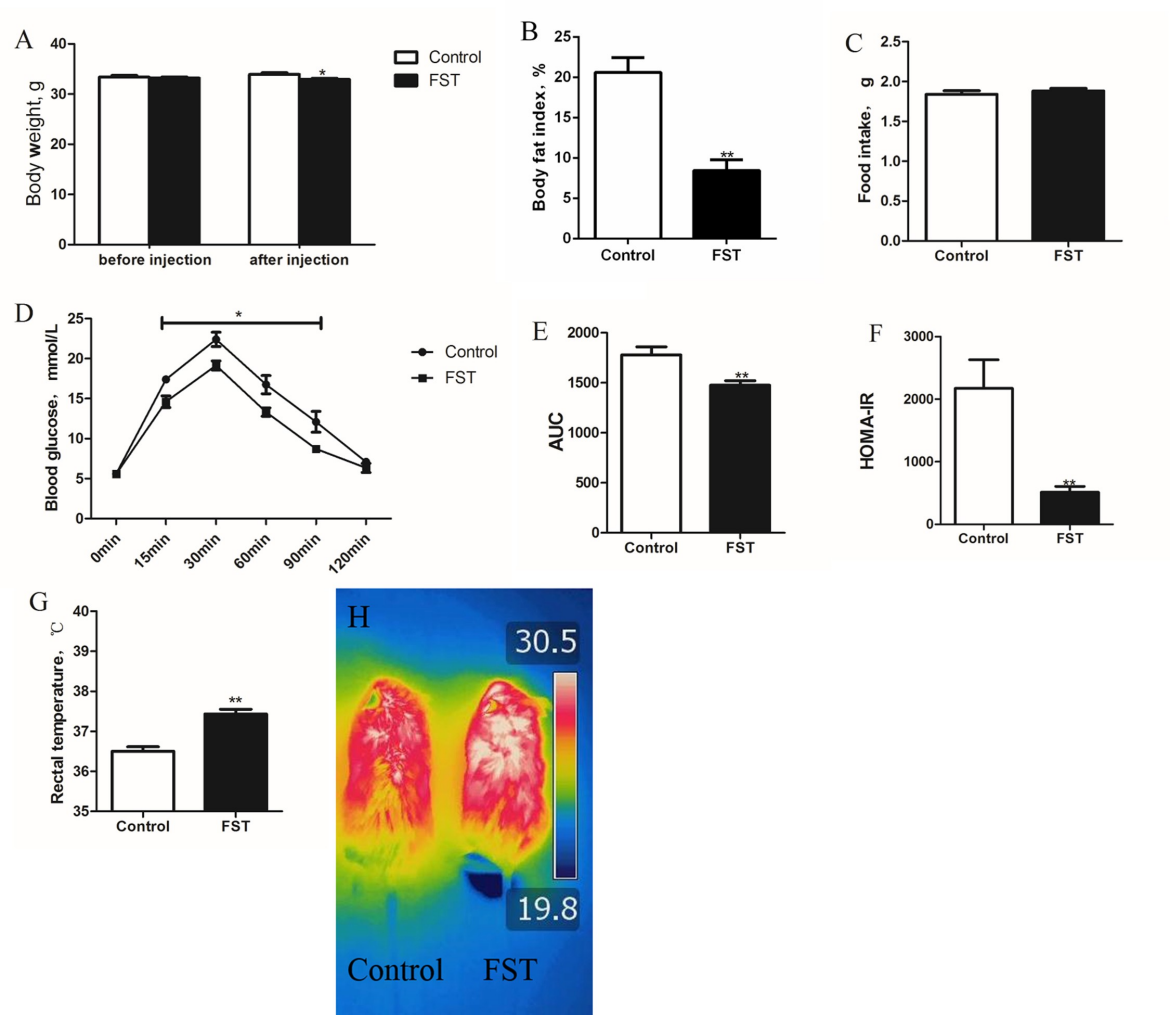
Effect of FST injection on body weight and blood glucose metabolism in DIO mice. A, FST injected mice lost weight after one week compared to the control group. B, FST injection decreased body fat index. C, No significant difference in food intake was observed between the FST injection group and the control group. D, GTT, FST injection increased the level of blood glucose metabolism in DIO mice. E, area under the curve of GTT. F, HOMA-IR, FST injection reduced insulin resistance in DIO mice. G-H, rectal temperature and infrared imagery of mice in the 4°C environment: FST injection group maintained a higher body temperature in the cold environment compared to the control group. Abbreviations: FST, the FST injection group, which received one-week injection of FST. Control, the control group, which had no treatment in the FST injection experiment. Bars represent the mean ± SEM, n = 6. *P < 0.05 and **P < 0.01 when the FST injection group was compared with the controls.

In the FST injection experiment, the high-fat diet-induced obese mice were intraperitoneally injected with FST once a day for one week. The body weight and body fat index were measured at the beginning and end of the experiment. The mice injected with FST had a lower body weight than the control group, which had no FST treatment (Fig 1A), and the body fat indexes of the FST injected mice were significantly lower than controls (Fig 1B). In addition, there was no significant difference in food intake, which was approximately 2 g high-fat chow per day, between the two groups (Fig 1C). The data suggest that FST injection can decrease body weight via a biological mechanism that does not inhibit food intake.

### FST injection improved glucose metabolism in obese mice

There was no significant difference in blood glucose between the two groups at 0 min. After glucose injection, the blood glucose of FST injected mice was significantly lower than that the control group at 15 min, 30 min, 60 min, 90 min (Fig 1D). The AUC of FST injected mice was significantly lower than the controls (Fig 1E). Compared to the control group, the mice injected with FST had a significantly lower HOMA-IR value (Fig 1F), which suggested that FST may be used to treat insulin resistance caused by obesity.

### FST injection improved thermogenesis in obese mice

After 4 h treatment at 4°C, the rectal temperature of the control group was approximately 36°C, while the rectal temperature of the FST injection group was approximately 37.5°C, which was significantly higher than the controls (Fig 1G). From the infrared imaging, the high-temperature area of the injection group was higher than that of the control group (Fig 1H). These results indicated that FST injection promoted thermogenesis in mice and allowed the maintenance of a higher body temperature under cold conditions.

### FST injection induced browning of subcutaneous adipocytes

**Fig 2:**
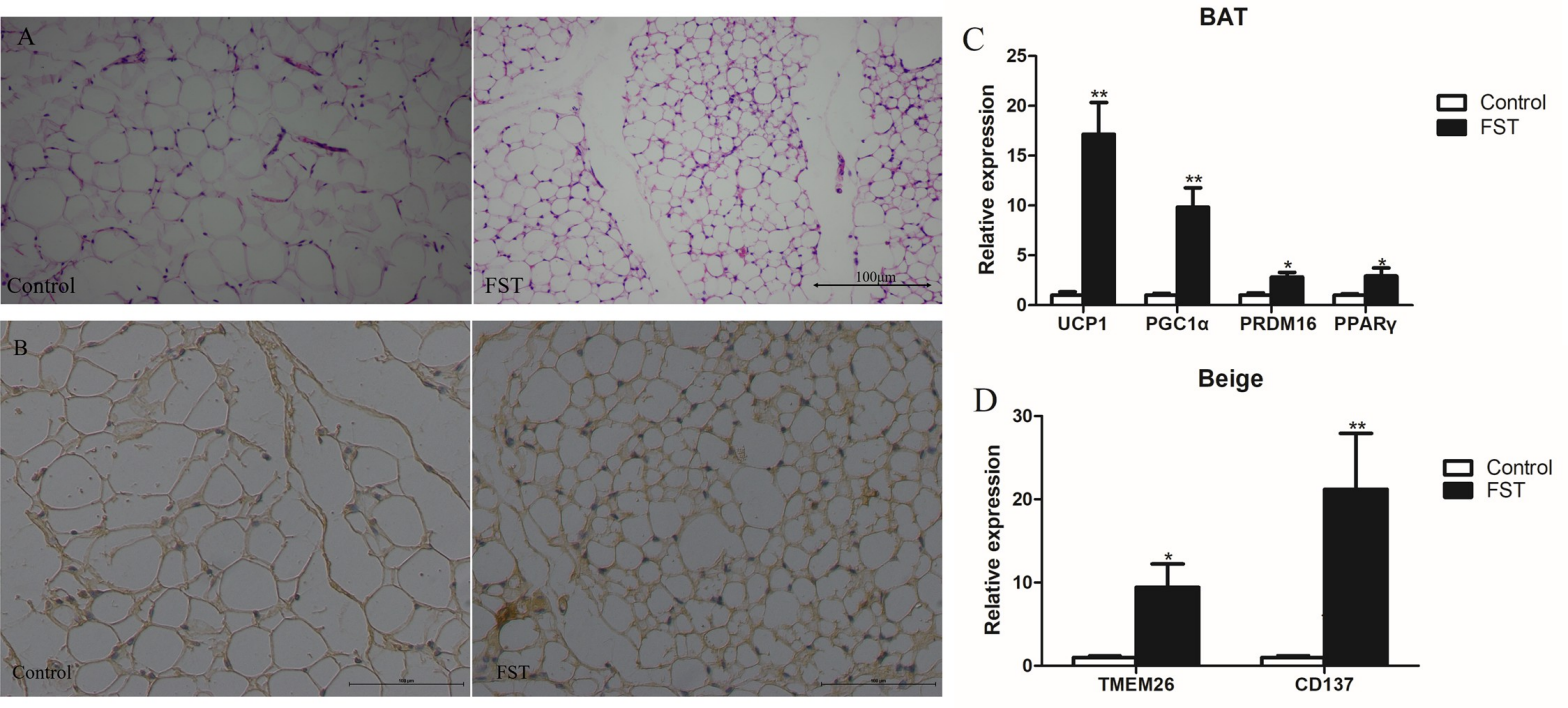
Effect of FST injection on the browning of inguinal subcutaneous WAT in DIO mice. A, hematoxylin and eosin staining of inguinal subcutaneous WAT taken from the two groups. After FST injection, the size of adipocytes was significantly reduced. B, IHC staining of Control and FST inguinal subcutaneous WAT using an anti-UCP1 antibody. C-D, the expression of BAT markers including *Ucp1*, *Pgc1α*, *Prdm16*, *Pparγ* and Beige markers including *Tmem26* and *Cd137*, injection of FST significantly increased the expression of these genes in the subcutaneous fat of DIO mice. Bars represent the mean ± SEM, n = 6. *P < 0.05 and **P < 0.01 when the FST injection group was compared with the control group. Abbreviations: FST, the FST injection group, which received one week of FST. Control, the control group, which had no treatment in the FST injection experiment.

By observing the H&E stained slides of the inguinal adipose under a microscope, it was determined that the size of subcutaneous adipocytes in FST injected mice was significantly smaller than in the controls, and multilocular lipid droplet structures appeared in the injection group (Fig 2A). Immunohistochemistry staining showed that the UCP1 signal in the FST group was stronger than the Control group (Fig 2B).

The expression levels of *Ucp1*, *Prdm16*, *Pgc1α* and *Pparγ* (the BAT maker genes involved in thermogenesis) in the injection group were significantly higher than in the control group (Fig 2C). The expression of *Tmem26* and *Cd137*, two marker genes of beige fat, in the injection group were significantly higher than in the control group (Fig 2D). Combined with characteristic gene expression and cell morphology, it can be concluded that FST injection induces subcutaneous WAT browning.

### Persistence of FST on physiological functions

**Fig 3:**
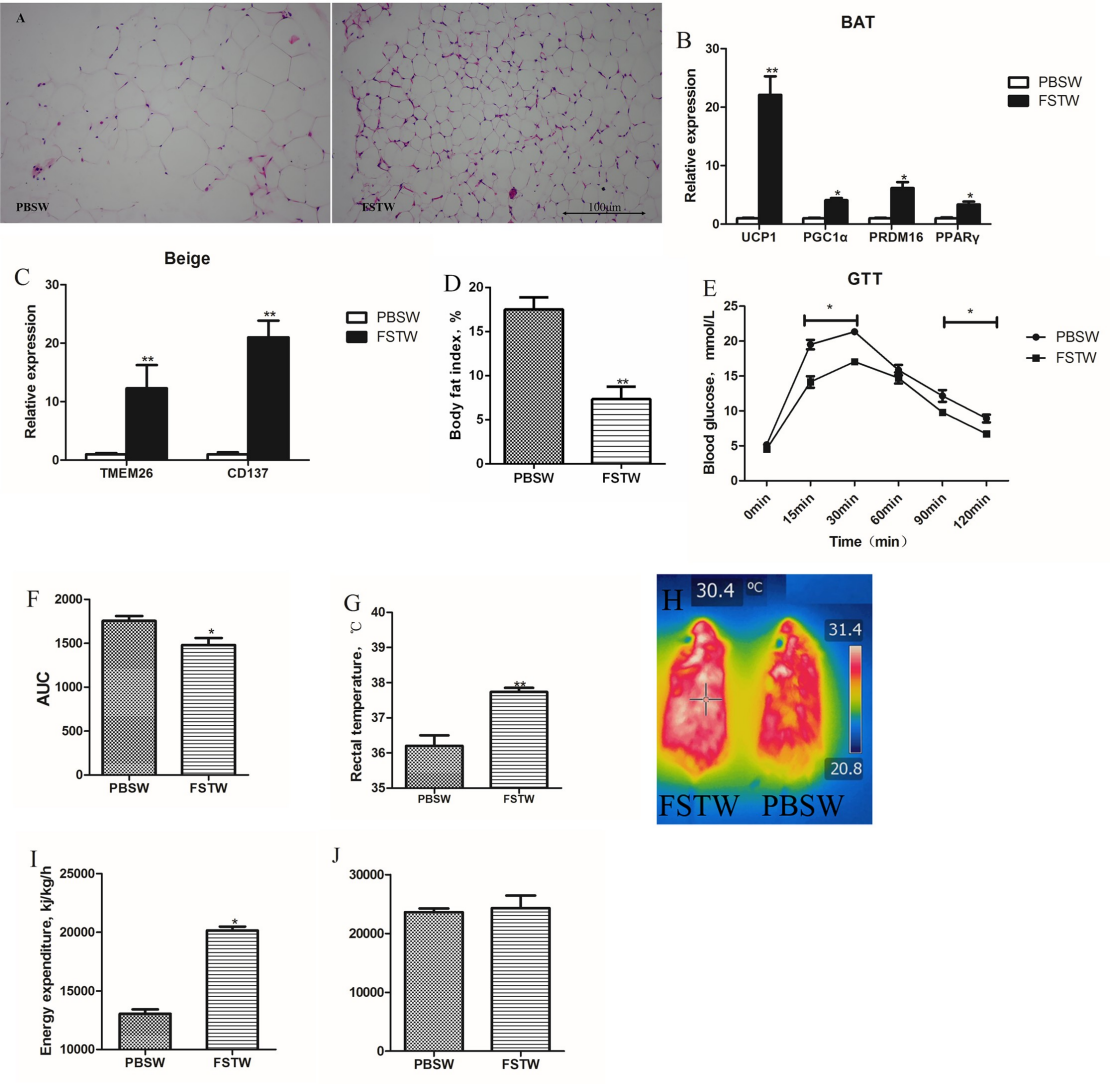
Persistence of FST on physiological function. A, hematoxylin and eosin staining of inguinal subcutaneous WAT taken from the FST withdrawal and PBS withdrawal groups. One week after FST withdrawal, the size of adipocytes in the FSTW group was still significantly smaller than in the PBSW group. B and C, the expression of BAT markers including *Ucp1*, *Pgc1α*, *Prdm16*, *Pparγ* and Beige markers including *Tmem26* and *Cd137*. After FST withdrawal, these key genes for the browning of WAT still maintained a high level of expression compared with the PBSW group. D, the body fat index of the FSTW group was significantly lower than in the PBSW group after FST withdrawal for one week. E and F, GTT and area under the curve of GTT, the level of blood glucose metabolism in the FSTW group was significantly higher than in the PBSW group one week after FST injection was withdrawn. G and H, rectal temperature and infrared imaging of mice in a 4°C environment. After stopping the FST treatment, the FSTW group still maintained a higher body temperature in a cold environment compared with the PBSW group, which served as the control. I and J, the energy expenditure and activity of mice in the FST withdrawal experiment. Bars represent the mean ± SEM, n = 6. *P < 0.05 and **P < 0.01 when the FST injection group was compared with the control. Abbreviations: FSTW (follistatin withdrawal), the group received one week of FST injections with no injections for the next week. PBSW (PBS withdrawal), the group received PBS injections for one week and no PBS injections for the next week.

Analysis of the pathological sections of the inguinal adipocyte tissue of mice showed that the size of subcutaneous adipocytes after stopping the drug was obviously smaller than the controls, which were injected with PBS for one week (Fig 3A). The expression of BAT markers and beige markers in subcutaneous adipocytes was detected by RT-qPCR and calculated using Cyclophilin as an internal control. The results showed that the expression of *Ucp1*, *Prdm16*, *Pgc1α* and *Pparγ* were significantly higher in the FST withdrawal group compared with the control group injected with PBS for a week (Fig 3B). Among these genes, UCP1 is a classical brown fat characteristic gene, and the expression of UCP1 in the FST withdrawal group was still increased by 22.1-fold compared with the controls (*p* < 0.01). *Tmem26* and *Cd137* are characteristic genes of beige fat, and the expression of these beige markers in the FST withdrawal group was significantly higher (increased by 12.3 and 21-fold, respectively) than in the PBS withdrawal group (Fig 3C).

To investigate the persistence of the physiological effects of FST, the FST withdrawal experiment was performed. The body fat rate of the mice in the FST withdrawal group was significantly lower than in the PBS withdrawal group (Fig 3D), even though the mice were still on a high-fat diet.

After the withdrawal of FST, a GTT was performed on the two groups. There was no significant difference in blood glucose between the groups at 0 min. At 15 min, 30 min, 90 min, and 120 min after glucose injection, the blood glucose of the mice in the FST withdrawal group was significantly lower than in the PBS withdrawal group (Fig 3E). The AUC results showed that the AUC values in the withdrawal group were significantly lower than the control either (Fig 3F).

After cold stimulation, the rectal temperature of the FST withdrawal group (about 37.5°C) was significantly higher than the control (about 36°C, Fig 3G). The high-temperature area in the FST withdrawal group was greater than in the controls (Fig 3H). To accurately measure the energy metabolism of the mice, the activity and oxygen consumption of the FSTW and the PBSW groups was measured using metabolic cages. The EE of the FSTW group was significantly higher than in the PBSW group (Fig 3I), although there was no significant difference in activity (Fig 3J).

Overall, FST increased the metabolic level and reduced the body weight of DIO mice, and this effect was maintained one week after cessation of FST. Considering that the inguinal subcutaneous fat was still beige fat after withdrawing FST, our data suggested that FST injection increased energy expenditure by promoting the browning of WAT.

### FST induced browning by promoting irisin secretion in beige adipose

**Fig 4:**
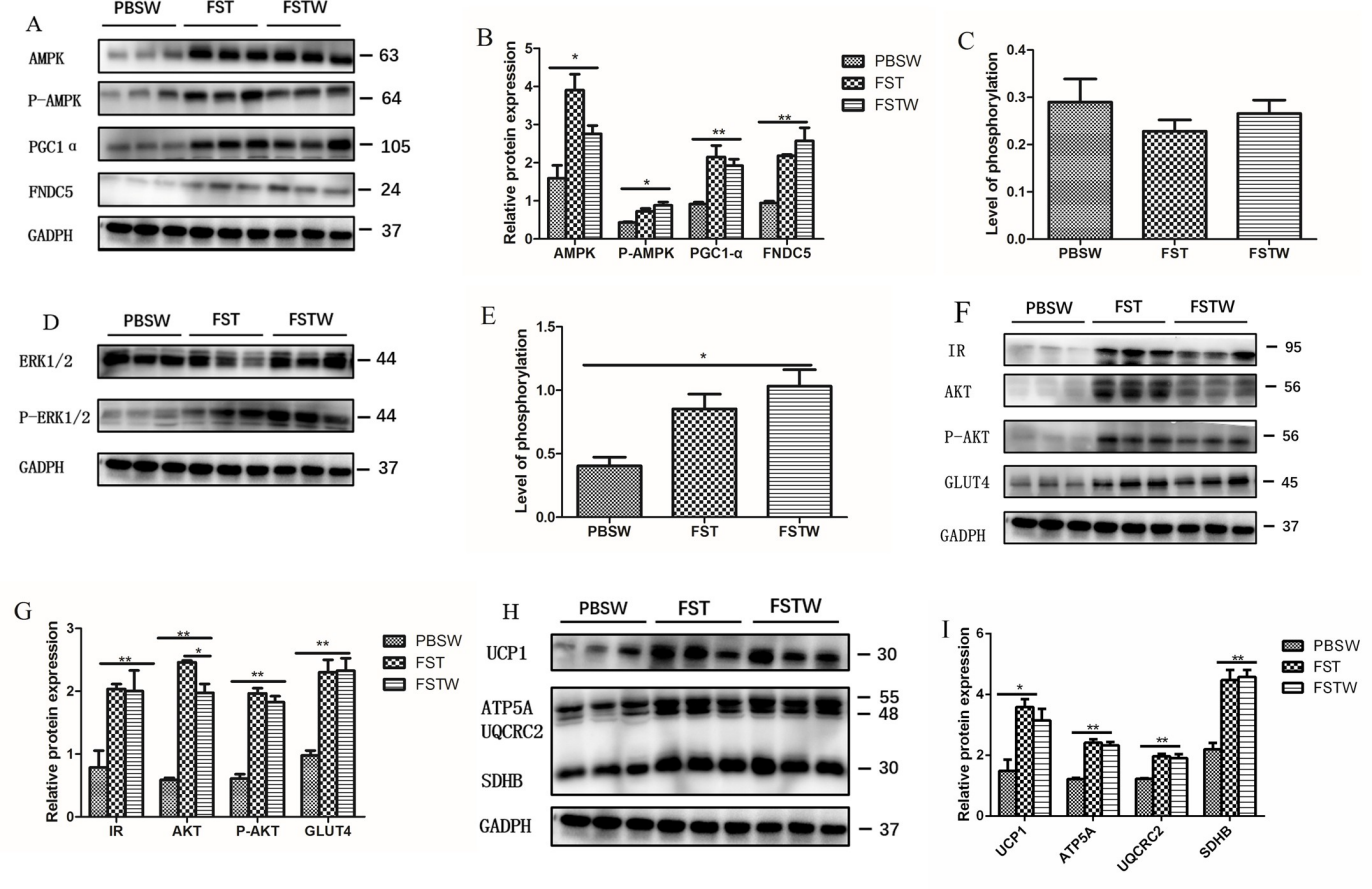
Western blotting of signaling pathway proteins following WAT browning and blood glucose metabolism in beige adipose. A, a representative western blotting of the AMPK-PGC1α-FNDC5 signaling pathway proteins. B, the expression levels of key proteins in the AMPK-PGC1α-FNDC5 pathway were significantly higher in the FSTW group compared with the PBSW group. C, the level of phosphorylation of AMPK. D, western blotting of ERK1/2 and phosphorylated ERK1/2. E, the phosphorylation level of ERK1/2 was significantly higher in the FSTW group compared with the PBSW group. F, western blotting of insulin signaling pathway proteins. G, the expression levels of IR, AKT, P-AKT and GLUT4 were significantly increased in the FSTW group compared with the PBSW group. H, western blotting of the proteins associated with mitochondrial activity. I, the expression levels of UCP1, ATP5A, UQCRC2, SDHB were significantly higher in the FSTW group compared with the PBSW group. Bars represent the mean ± SEM, n = 6. *p < 0.05 and **p < 0.01 when the FST injection group was compared with the controls. Abbreviations: PBSW (PBS withdrawal), the group received PBS injection for one week and no PBS injection for the next week. FST, the group received one week of follistatin injection and was then sacrificed in the FST withdrawal experiment (independent of the FST group in the FST injection experiment). FSTW (follistatin withdrawal), the group received one week of FST injection and no injections for the next week.

The expression of AMPK, p-AMPK, PGC1α and FNDC5 in the FST withdrawal group was significantly higher than in the PBS withdrawal group (Fig 4A and 4B), and the FNDC5 protein level was 2.7-fold higher (*p* < 0.01, Fig 4A and 4B). It is worth noting that there was no significant difference in the four proteins between the FST group and the FST withdrawal group (Fig 4A and 4B). These results suggested that FST may induce browning of subcutaneous adipose by promoting the secretion of irisin in subcutaneous adipose. In addition, although the expression levels of AMPK and p-AMPK increased significantly after injection of FST, the phosphorylation level of AMPK did not change significantly (Fig 4C).

To further verify that FST induced subcutaneous fat browning through the irisin signaling pathway, the phosphorylation levels of ERK1/2, the downstream signal of irisin, was detected. The phosphorylation level of ERK1/2 pathway proteins in the FST withdrawal group was increased by 2.11-fold (*p* < 0.05) compared to the PBS withdrawal group (Fig 4D and 4E). Interestingly, there was no significant difference between the FST and FST withdrawal groups (Fig 4D and 4E).

### FST promoted blood glucose metabolism by activating the insulin signaling pathway in beige adipose

The expression IR, AKT, P-AKT, and GLUT4, four key proteins in the insulin signaling pathway, were measured in the FST, FST withdrawal and PBS withdrawal groups.

The expression of key proteins in the insulin signaling pathway in the FST withdrawal group was significantly higher than in the PBS withdrawal group. The expression of IR was increased by 2.5-fold (*p* < 0.01), AKT was increased by 3.4-fold (*p* < 0.01), P-AKT was increased by 3.0-fold (*p* < 0.01) and Glut4 was increased by 2.4-fold (*p* < 0.01) compared to PBS withdrawal group. Except for the AKT protein, there was no significant difference between the FST group and the FST withdrawal group in the insulin signaling pathway proteins (Fig 4F and 4G).

### FST promotes mitochondrial heat production in adipocytes

The increased thermogenesis in mice injected with FST was associated with mitochondrial activity in beige fat. The results of western blotting showed that the expression of key proteins in the mitochondria of the FST withdrawal group was significantly higher than in PBS withdrawal group. The expression of UCP1 was increased by 2.1-fold (*p* < 0.01) compared to the PBS withdrawal group. The increase in expression of UCP1 and OXPHOS including ATP5A, UQCRC2 and SDHB, suggested that the level of phosphorylation and uncoupling in mitochondria was promoted by FST. There was no significant difference between FST group and FST withdrawal group on these mitochondrial proteins, which resulted in an intensification of energy metabolism in the mice (Fig 4H and 4I).

## Discussion

Browning of WAT, as one of the important biological functions of FST, has been a focus of current research. FST injection groups exhibited a browning phenotype with respect to cell morphology after FST injection, and the size of the adipocytes was significantly reduced. The heat-producing function of beige fat and brown fat is largely dependent on the uncoupling effect of UCP1, a typical brown fat characteristic gene. It is an important reference for determining adipose browning. In this experiment, the expression of *Ucp1*, *Prdm16*, *Pgc1α*, and *Pparγ* was significantly increased after injection of FST. Aside from the brown fat characteristic genes, the beige fat characteristic genes are an important criterion for judging WAT browning. Garron Dodd *et al*. used leptin and insulin to promote fat browning, and found that adipocytes shrank and the expression of brown markers and beige markers increased. J Lin et al. also showed similar results in experiments with Tibetan pigs, in which the browning was attributed to UCP3 which is an uncoupling protein similar to UCP1 [27, 28]. In our study, the expression of *Tmem26* and *Cd137*, two beige markers, increased after injection of FST, indicating that FST injection induced inguinal subcutaneous WAT browning in obese mice.

Obesity caused by a high-fat diet was significantly suppressed following injection with FST. The body weights and body fat indexes of mice injected with FST were significantly reduced without changing food intake. S Fang *et al*. used FXR agonists to induce browning of WAT in mice. Their results showed a similar phenotype as in the current study, in that there was weight loss after WAT browning in mice [29]. Taken together, these results suggest that FST may inhibit obesity through beige fat generation.

One of the important functions of beige fat is to promote heat production and increase energy metabolism. After the browning of subcutaneous adipose, the WAT (which serves as an energy storage tissue) became beige adipose tissue (which serves as an energy consumption tissue and is directly related to the activation of mitochondrial function and an increase in the number of mitochondria in adipose) [30]. UCP1, the core protein of uncoupling, is key to the function of brown fat and beige fat, and plays an important role in energy release [31]. In the UCP1 overexpressing transgenic mouse model, mice were resistant to obesity induced by a high-fat diet [32]. Pontus *et al*. successfully constructed a mouse model which overexpressed PGC1α. The transgenic mice showed browning of subcutaneous WAT, and UCP1 protein content was significantly increased in browned beige fat, results that are similar to the current experiment using FST injection [33]. These results indicated that FST promoted thermogenesis by promoting the expression of UCP1 in beige fat and increasing the uncoupling level of mitochondria. In FST knockout mouse embryonic fibroblasts, cell oxygen consumption was significantly lower than in wild-type mouse embryonic fibroblasts, and beige fat exhibited considerable heat production due to the large amount of UCP1. [30, 34]. In the current experiment, the thermogenesis of mice was increased after FST injection (Fig 1G and 1H), and the expression of the *Ucp1* gene in beige fat was increased (Fig 2C), indicating that FST resists obesity by promoting energy metabolism in beige fat.

Type 2 diabetes is a classic metabolic disease caused by obesity. In this study, after the injection of FST, blood glucose tolerance increased and insulin resistance decreased, indicating that FST improved blood glucose metabolism in obese mice. Many experiments have shown that when MST is inhibited, adipocytes are browned. As an antagonist of MST, FST can promote the expression of the PGC1α gene, a classical BAT marker, when it acts on adipose cells [30, 35]. In the MST knockdown pig model constructed by Chunbo Cai *et al*. and the PGC1α overexpressed mouse model constructed by Pontus Bostrom *et al*., insulin sensitivity in experimental animals was up-regulated with fat browning [33, 36]. In experiments on blood glucose metabolism, studies have generally focused on the muscles. In the gastrocnemius muscle of MST knock out Meishan pigs, the expression level of the insulin signaling pathway IR-AKT-GLUT4 was significantly increased compared to that of wild type pigs [36]. Beige fat, as an energy metabolism tissue, also plays an important role in blood glucose metabolism. In our study, western blot analysis of subcutaneous fat revealed that the insulin signaling pathway proteins in beige fat were significantly improved (Fig 4F and 4G), indicating that FST can regulate blood glucose by activating the insulin pathway in fat as well as muscle.

Irisin is a recently discovered muscle factor which promotes WAT browning and regulates blood glucose. Blood irisin content is sharply increased after vigorous exercise, suggesting that exercise promotes skeletal muscle secretion of irisin. In the myocytes of MST knock out mice, the amount of irisin secretion was significantly higher than in the wild type mice. In the experiments on MST knockout animal models performed by Chunbo Cai *et al*., MST deletion promoted the secretion of irisin via the AMPK-PGC1α-FNDC5 signaling pathway [36]. Roca-Rivada *et al*. found that adipocytes also secrete irisin, suggesting that similar to muscles, the irisin secretion may be present in adipose tissue [37]. In the current experiment, the AMPK-PGC1α-FNDC5 pathway in beige fat was verified. The protein expression level in the FST withdrawal group was significantly higher than in the PBS withdrawal control group. This suggested that FST regulated the synthesis of irisin in beige fat though promoting AMPK and PGC1α expression (Fig 4A and 4B). Yuan Zhang *et al*. reported that irisin promoted the browning of 3T3L1 adipocytes when U0126 inhibitors were used to block the ERK1/2 phosphorylation pathway. In this experiment, adipocytes did not turn beige, suggesting that irisin induces browning via the ERK1/2 signal pathway. The results of the current experiment showed that the phosphorylation levels of ERK1/2 signaling pathway proteins was significantly increased after FST injection in obese mice. It can be speculated that after the FST injection, the secretion of irisin is promoted by the AMPK-PGC1α-FNDC5 pathway in muscle and fat. Irisin induces browning of subcutaneous fat by promoting the phosphorylation levels of ERK1/2 (Fig 4A–4E). Other studies have shown that the concentration of blood irisin is inversely proportional to insulin sensitivity, that is, when insulin resistance occurs in the body, the blood concentration of irisin is also significantly increased. This indicates that FST can increase insulin sensitivity by promoting the secretion of irisin.

Many different drugs are available and induce effect of weight reduction via suppressing appetite, promoting excretion, and hindering absorption, such as bupropion, L-carnitine, and orlistat [38–40]. Due to the influence of drug mechanisms, most drugs cause side effects such as diarrhea, fecal incontinence, depression, or addiction. People must also continue to take the medicines to maintain weight loss, and a rebound in weight gain can also occur if people withdraw the medicines. Therefore, most of these small molecules have been banned [41]. In the FST withdrawal experiment, after one week of FST withdrawal, the effect of FST on the inhibition in fat increase, promotion of body heat production, and acceleration of blood glucose metabolism did not disappear (Fig 3D–3J), indicating that the effects of FST are persistent.

FST injection increased the metabolism of beige fat, and the expression of AKT was slightly decreased. Except for AKT, the expression of insulin pathway proteins did not change significantly after cessation of FST treatment (Fig 4F and 4G). FST promoted expression of the UCP1 and OXPHOS proteins in the mitochondria of beige fat, which aggravated the uncoupling and oxidative phosphorylation of adipocytes. In addition, the relative expression of UCP1 and OXPHOS did not decrease significantly after one week of cessation of FST injection. This may explain how metabolism promotion in obese mice can still exist even if the FST is withdrawn.

## Conclusions

Due to the development of social productivity, obesity and related metabolic diseases induced by high-fat diets and a sedentary lifestyle are plaguing people at a rapid pace. Our study suggests that FST, an autocrine and paracrine protein, has great potential in the treatment of obesity and metabolic syndrome by inducing the browning of white fat and promoting glucose and lipid metabolism. FST may therefore be developed as a new drug for the treatment of obesity. However, further evaluation of safety and the physiological actions of FST require more exploration.
