# Correction: Intraperitoneal administration of follistatin promotes adipocyte browning in high-fat diet-induced obese mice

**DOI:** 10.1371/journal.pone.0226344

**Published:** 2019-12-05

**Authors:** Haoyu Li, Chuanhai Zhang, Junyu Liu, Wenya Xie, Wentao Xu, Fei Liang, Kunlun Huang, Xiaoyun He

The authors acknowledge the following incorrectly duplicated references:

Reference [3] also appears as reference [31]. The reference is: Nedergaard J, Bengtsson T, Cannon B. New Powers of Brown Fat: Fighting the Metabolic Syndrome. Cell Metabolism. 2011;13(3):238–40.

Reference [9] also appears as references [16] and [26]. The reference is: Singh R, Braga M, Reddy ST, Lee SJ, Parveen M, Grijalva V, et al. Follistatin targets distinct pathways to promote brown adipocyte characteristics in brown and white adipose tissues. Endocrinology. 2017;158(5):1217–30.

Reference [12] also appears as reference [24]. The reference is: Shan T, Liang X, Bi P, Kuang S. Myostatin knockout drives browning of white adipose tissue through activating the AMPK-PGC1α-Fndc5 pathway in muscle. The FASEB Journal. 2013;27(5):1981–9.

Reference [11] also appears as reference [35]. The reference is: Braga M, Pervin S, Norris K, Bhasin S, Singh R. Inhibition of in vitro and in vivo brown fat differentiation program by myostatin. Obesity. 2013;21(6):1180–8.

Reference [15] also appears as reference [20]. The reference is: Braga M, Reddy ST, Vergnes L, Pervin S, Grijalva V, Stout D, et al. Follistatin promotes adipocyte differentiation, browning, and energy metabolism. Journal of Lipid Research. 2014;55(3):375–84.

Reference [17] also appears as references [25] and [33]. The reference is: Boström P, Wu J, Jedrychowski MP, Korde A, Ye L, Lo JC, et al. A PGC1-α-dependent myokine that drives brown-fat-like development of white fat and thermogenesis. Nature. 2012;481(7382):463–8.

Reference [18] also appears as reference [37]. The reference is: Arturo RR, Cecilia C, Senin LL, Landrove MO, Javier B, Ana BC, et al. FNDC5/irisin is not only a myokine but also an adipokine. Plos One. 2013;8(4):e60563.

In addition, there are typographical errors in the following references:

In reference [17], “α” incorrectly appears as “[agr].” The correct reference is: The reference is: Boström P, Wu J, Jedrychowski MP, Korde A, Ye L, Lo JC, et al. A PGC1-α-dependent myokine that drives brown-fat-like development of white fat and thermogenesis. Nature. 2012;481(7382):463–8.

In reference [19], “SAT-123” incorrectly appears as “SAT-123-SAT-.” The correct reference is: Triantafyllou GA, Skouvaklidou EC, Saridakis ZG, Kynigopoulos G, Dima DI, Apostolou A, et al. Circulating Follistatin and Irisin in Young, Healthy Individuals: A One-Year Prospective Cohort Study. GPCRs, Growth Factors, Tyrosine Kinases, Inhibits, Activins, and TGF Beta Superfamily (posters): Endocrine Society; 2016. p. SAT-123.

In reference [25], “brown-fat-like development” incorrectly appears as “browning.” The correct reference is: Boström P, Wu J, Jedrychowski MP, Korde A, Ye L, Lo JC, et al. A PGC1-α-dependent myokine that drives brown-fat-like development of white fat and thermogenesis. Nature. 2012;481(7382):463–8.

Reference [33] contains multiple errors. “Boström” incorrectly appears as “Bostrã M.” “PGC1-Î±-dependent” should appear as “PGC1-α-dependent.” The correct reference is: Boström P, Wu J, Jedrychowski MP, Korde A, Ye L, Lo JC, et al. A PGC1-α-dependent myokine that drives brown-fat-like development of white fat and thermogenesis. Nature. 2012;481(7382):463–8.

In reference [35], “ofin vitroandin vivobrown” should appear as “of in vitro and in vivo brown.” The correct reference is: Braga M, Pervin S, Norris K, Bhasin S, Singh R. Inhibition of in vitro and in vivo brown fat differentiation program by myostatin. Obesity. 2013;21(6):1180–8.
